# EFFECTS OF A SITTING REDUCTION INTERVENTION FOR OBESE OLDER ADULTS WITH DEPRESSION

**DOI:** 10.1093/geroni/igz038.1921

**Published:** 2019-11-08

**Authors:** Sarita A Hemmady, Dori Rosenberg, Peggy Hannon, Jing Zhou

**Affiliations:** 1 University of Washington, Seattle, Washington, United States; 2 Kaiser Permanente Washington Health Research Institute, Seattle, Washington, United States

## Abstract

Background: Little is known about the impact of sedentary behavior (SB) reduction interventions on older adults with obesity and depressed mood. An exploratory analysis examined behavioral and mental health effects of a SB reduction among participants with depressed moods. Methods: Participants were obese older adults (n=30, mean age=66, 77% female, 23% male, mean PHQ-8-Score=13.67) that were randomized to receive a sitting reduction intervention ( I-STAND); N=16) or a control condition (N=14) as part of a larger trial. Participants wore activPAL devices to assess sitting time at baseline and 12-weeks; they also completed the Patient-Health Questionnaire-8 (PHQ-8) to assess depressive symptoms. Linear regression models compared baseline and 12-week measures between groups adjusting for baseline values. A post-hoc qualitative analysis assessed ISTAND participant interview data. Results: I-STAND participants had greater reductions in sitting time than control participants by 57-minutes (p=0.04), as well as greater reductions in percent sitting time by 5.89-percent (p=0.03). Mean PHQ-8 scores were decreased by 0.14-points among the I-STAND group compared to the control (P=0.90). Qualitative themes included physical and social barriers to standing; varying perceptions of the presence of depression; physical health improvements (i.e. mood improvement) and perceptions of physical activity (i.e. feasibility to exercise). Conclusion: We found significant associations between sitting reduction and a SB intervention among older adults with obesity and depression, however this did not impact depressive symptoms. Further research should examine whether sitting reduction can improve mood or standing time among older adults with obesity and depressed mood.

